# Trait Emotional Empathy and Resting State Functional Connectivity in Default Mode, Salience, and Central Executive Networks

**DOI:** 10.3390/brainsci8070128

**Published:** 2018-07-06

**Authors:** Elena Bilevicius, Tiffany A. Kolesar, Stephen D. Smith, Paul D. Trapnell, Jennifer Kornelsen

**Affiliations:** 1 Department of Physiology and Pathophysiology, University of Manitoba, Winnipeg, MB R3E 0J9, Canada; bilevice@myumanitoba.ca (E.B.); tiffany.kolesar@umanitoba.ca (T.A.K.); 2 St. Boniface Hospital Research, Catholic Health Corporation of Manitoba, Compassion Project, Winnipeg, MB R2H 2A6, Canada; 3 Department of Psychology, University of Manitoba, Winnipeg, MB R3T 2N2, Canada; 4 Department of Psychology, University of Winnipeg, Winnipeg, MB R3B 2E9, Canada; s.smith@uwinnipeg.ca (S.D.S.); paultrapnell@gmail.com (P.D.T.); 5 Department of Radiology, University of Manitoba, Winnipeg, MB R3A 1R9, Canada

**Keywords:** emotional empathy, resting state, functional connectivity, functional magnetic resonance imaging

## Abstract

Emotional empathy is the ability to experience and/or share another person’s emotional states and responses. Although some research has examined the neural correlates of emotional empathy, there has been little research investigating whether this component of empathy is related to the functional connectivity of resting state networks in the brain. In the current study, 32 participants answered a trait emotional empathy questionnaire in a session previous to their functional magnetic resonance imaging scan. Results indicate that emotional empathy scores were correlated with different patterns of functional connectivity in the default mode network (DMN), salience network (SN), and left and right central executive networks. For example, within the DMN, emotional empathy scores positively correlated with connectivity in the premotor cortex. Within the SN, empathy scores were positively correlated with the fusiform gyrus and cuneus. These findings demonstrate that emotional empathy is associated with unique patterns of functional connectivity in four of the brain’s resting state networks.

## 1. Trait Emotional Empathy and Resting State Functional Connectivity in Default Mode, Salience, and Central Executive Networks

Empathy is a multifaceted construct consisting of the ability to identify and understand another person’s perspective [1]. This ability involves two distinct facets: a cognitive component, which conceptualizes empathy as a cognitive process whereby an individual imagines a situation from another perspective, and an emotional component, thought of as the shared emotional experience with the observed other [2]. Together, these facets of empathy greatly facilitate one’s ability to understand human emotions and social behavior [1,3].

In its most simplistic state, empathy provides warmth and nurturance to increase the chance of survival in offspring [4]. Evolutionary psychologists have taken this idea one step further, suggesting that viewing or thinking of someone in an emotional state will activate the same brain regions in the observer as it will in the individual experiencing the emotional state first-hand [5].

Functional magnetic resonance imaging (fMRI) experiments have identified numerous brain regions involved with both cognitive and emotional empathy. Many earlier studies highlighted the role of the posterior inferior frontal gyrus (IFG) and posterior parietal cortex in both cognitive and emotional (e.g., [6,7,8]) empathy. Later studies of empathy have attempted to identify differences in the neural activity associated with cognitive and emotional empathy. For instance, Nummenmaa and colleagues [9] found that emotional empathy was associated with increased activity in the fusiform gyrus, parahippocampal gyrus, postcentral gyrus, insula, middle occipital gyrus, and premotor cortex. Cognitive empathy, on the other hand, led to greater activity in cortical systems related to mentalizing and theory of mind including the middle frontal sulcus, cuneus, parahippocampal gyrus, and fusiform gyrus. These results are complemented by a lesion study in which impairments in cognitive empathy—particularly theory of mind abilities—were associated with ventromedial prefrontal cortex lesions whereas impairments in emotional empathy were caused by IFG lesions (Brodmann area [BA] 44) [10]. Although some overlap exists between the neural correlates of cognitive and emotional empathy, these studies also suggest that some of the neural underpinnings of emotional empathy are distinct from cognitive empathy.

Empathy can be thought of as a state (i.e., empathy in the current moment) or a trait (i.e., a stable, intrinsic trait). As in other emotions, empathy is variable from person-to-person, and people have different baseline levels of empathy. For the purposes of the current study, we will refer to this as “trait empathy”. Trait empathy is commonly assessed through self-report measures and can quantify both cognitive and emotional facets of empathy [11]. Although both trait emotional and cognitive empathy are important, there is evidence suggesting that deficits in emotional, but not cognitive empathy, are associated with alcoholism [12] and social anxiety disorder [13]. This dissociation suggests there is something unique about emotional empathy that is important to explore further.

Recently, Kim and colleagues [14] used resting state fMRI to look at how trait empathy relates to brain function. Resting state fMRI detects functional connectivity; that is, correlated fluctuations in neural activity in disparate brain regions that are measured when the participant is not performing a cognitive or attentional task (i.e., is “at rest”) [15,16,17]. Kim and colleague’s study revealed reduced functional connectivity in the medial prefrontal and anterior cingulate cortices in people who are lower than average in empathy. The two brain regions identified are associated with two commonly studied resting state networks—the default mode network (DMN) and salience network (SN).

The DMN—which includes the medial prefrontal cortex, lateral parietal cortex, and precuneus/posterior cingulate cortex—shows pronounced activity when an individual is not attending to an external stimulus [15,17,18]. The DMN is thought to be involved in internally focused and self-referential mentation [19,20], behaviors necessary for empathy (e.g., [8]). The insula and anterior cingulate cortex make up the SN, which is thought to be involved with sensitivity to external stimuli and the integration of sensory information [21,22]. To show emotional empathy, one must be able to recognize another individual’s emotional experience and reflect that inward [10]. This process should influence one’s internal physical states, which in turn should correlate with activity in the neural structures that make up the SN [23,24]. Additional resting state networks of potential relevance to trait empathy are the left and right central executive networks (CEN); involved in information processing and decision making [21,25]. Typically appearing as statistically distinct networks, they both consist of unilateral regions of the dorsolateral prefrontal cortex and dorsal parietal lobes. To date, there have been few examinations of trait emotional empathy using resting state fMRI but none using a whole-brain, data-driven analysis [11,14]. In the current study, we tested resting state functional connectivity using a whole-brain, data-driven method to determine whether measures of functional connectivity in the DMN, SN, and left and right CEN related to individual differences in trait emotional empathy. These data will further our understanding of the neural basis of emotional empathy by providing novel information about how disparate regions of the brain work together to produce a socially important personality trait.

## 2. Method

### 2.1. Participants

Thirty-two students from an Introduction to Psychology course at the University of Winnipeg participated in the study (10 males, 22 females; mean age = 18.16; SD = 1.08; range 17–22 years). The education level of the participants was quite homogeneous, with all participants being first-or second-year university students. Exclusion criteria for this study included the presence of metal in the body and/or a history of diagnosed psychiatric illness or neurological injury. All participants provided informed consent before beginning the study and received a $25 honorarium for their participation. The study was approved by the institutional ethics review boards of both the University of Winnipeg and the National Research Council.

### 2.2. Empathy Questionnaire

The empathy measure consisted of six trait descriptive verbal phrases, five of which are from the International Personality Item Pool (IPIP, [26]). The verbal phrase format of IPIP items allowed these empathy items to be embedded in a popular research measure of the so-called Big Five personality trait dimensions (the Big Five Inventory [BFI]; see below). Five items were selected from the IPIP on the basis of face validity with respect to leading self-report questionnaire measures of empathy, such as the Empathic Concern subscale of the Interpersonal Reactivity Index [1] and the Compassionate Love Scale [27]. Participants rated these items for self-descriptiveness using the BFI instructions and response format. Third person wordings were used in order to match the format of BFI items. The six empathy items were: (1) Feels others’ emotions; (2) Suffers from others’ sorrows; (3) Anticipates the needs of others; (4) Is deeply moved by others’ misfortunes; (5) Senses others’ wishes; and (6) Is very empathic, intensely feels what others feel. Items 1–5 are IPIP items. Item 6 was a phrase adaptation of two empathy items from another measure [28]. Cronbach’s Alpha for this measure (based on a sample of 962 Introductory Psychology students from a larger study related to personality traits, including the 32 participants who were scanned) was reasonably high: α = 0.78; the Cronbach’s Alpha for the 32 participants in our study was α = 0.84. Additional information about the psychometric properties of the empathy questionnaire are included as Supplementary Material.

### 2.3. Procedure

The experimental procedure involved two sessions. In the first session, participants completed a measure of empathy as part of a larger study of personality traits. The second session of the experiment involved neuroimaging. Participants underwent MRI safety screening in order to ensure that they could safely enter the MRI scanner. To minimize motion, stiff foam pads were placed between the participants’ head and the inside of the head coil. High-resolution 3D anatomical and resting state functional MRI data were then acquired for each participant. Participants were instructed to remain awake and lay still with their eyes closed throughout the duration of the scan, and all participants reported they were able to remain awake.

### 2.4. Data Acquisition

Data were acquired using a 3-Tesla Siemens TRIO MRI scanner (Siemens, Erlangen, Germany). The high resolution anatomical data were collected using an MP-RAGE sequence (1 mm slice thickness, 0 gap, TR/TE = 1900/2.2 ms, 256 × 256 matrix, field of view [FOV] = 24 cm). A whole-brain echo planar imaging (EPI) sequence was used to acquire the functional data in 140 slices (0 gap, TR/TE = 3000/30 ms, flip angle = 90°, 64 × 64 matrix, FOV 24 cm).

### 2.5. Data Analysis

Preprocessing. All data preprocessing and statistical analyses were completed using BrainVoyager QX 2.8.4 software (Brain Innovation BV, Maastricht, The Netherlands). The preprocessing of functional data included slice scan time correction using cubic spline interpolation, a 3D motion correction in the six possible directions using a trilinear/sync interpolation, a GLM-Fourier temporal filtering at 2 sines/cosines and 8-mm full-width half-maximum Gaussian spatial smoothing. Motion was regressed out from the data before moving on to further analyses. The preprocessed functional data were co-registered to anatomical data in native space. Anatomical data was spatially normalized by warping the data into standardized Talairach space. The anatomical and co-registered functional data were then used to warp the functional time course data into Talairach space.

Independent Component Analysis. An independent component analysis (ICA) was run for all 32 participants to detect the correlated neural activity fluctuations within each functional time series. A FastICA algorithm was used with a deflation approach to produce 20 independent components (ICs) per participant. The FastICA algorithm first extracts the components with highly nonGuassian distributions (likely to be neurologically meaningful patterns) and components with the most Guassian distributions (likely to be spatially distributed noise) last. Each individual-level set of ICs were then run through a self-organizing group-level ICA BrainVoyager plugin (SogICA; [29]). The SogICA first computes a linear correlation coefficient between maps to measure similarity between ICs. These values are converted to Euclidean distances which are put into a matrix. Next, a hierarchical clustering procedure groups the ICs based on the distance matrix. Only one IC per participant is entered during the creation of each group-level component, resulting in a set of 20 components composed of highly similar IC’s across participants [29]. As a result of the hierarchical clustering, the final set of group-level ICs contain robust resting state networks as well as noise components [29]. No template or mask was applied to the maps—the functional connectivity within the ICs represents a voxel-to-voxel analysis that is not restricted to connectivity between a priori seeds or regions-of-interest. Following the SogICA, the resting state networks were visually inspected and cross-referenced with verified Talairach coordinates [30,31] to identify the DMN, SN, and left and right CEN. A figure of the ICs identified for each network and a table listing the coordinates have been previously published [32].

ANCOVA. In order to determine how IPIP scores co-vary with the functional connectivity within the resting state networks, each participant’s overall scores from the questionnaire were entered as covariates in an analysis of covariance (ANCOVA) for each of the four identified ICs (i.e., within the ICs showing the DMN, SN, and left and right CEN). The resulting brain maps show the correlation between the IPIP scores and the average time series in every voxel in each of the four maps. The maps, displayed at *p* < 0.05, were corrected for multiple comparisons using a cluster threshold estimator, derived from Monte Carlo simulations (1000 iterations) [33,34]. The clusters exceeding the threshold within each corrected map were converted to volumes-of-interest. The Talairach coordinates for the peak voxel of each cluster the *r*-values, and cluster sizes were extracted for each cluster. Talairach coordinates were exported and run through Talairach-Daemon software (http://www.talairach.org/daemon.html) to provide the region and Brodmann area (BA), if applicable.

## 3. Results

**Figure 1:**
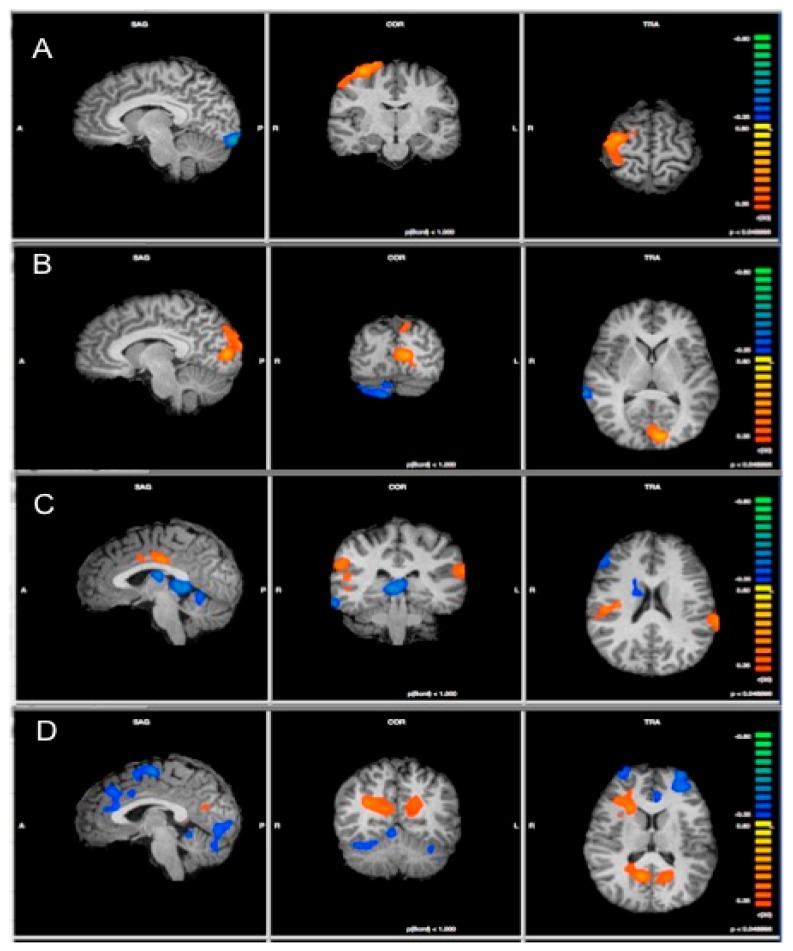
Results of analysis of covariance (ANCOVA) showing regions of significant correlation between trait emotional empathy scores and the functional connectivity within each of the four components representing the networks: (**A**) Default Mode; (**B**) Salience; (**C**) Left Central Executive, and (**D**) Right Central Executive network. Positive correlations are indicated in orange while negative correlations are indicated in blue. Maps are displayed at *p* < 0.05 with a cluster threshold estimator applied to reduce false positives.

Functional connectivity within the DMN, SN, left CEN, and right CEN ICs was correlated with trait emotional empathy scores. Detailed results can be found in Table 1 and Figure 1, and are described by network below. Clusters that have a positive *r*-value indicate that IPIP scores were positively correlated with the functional connectivity in that cluster, whereas clusters with a negative *r*-value indicate that IPIP scores are negatively correlated with the functional connectivity in that cluster. Importantly, the brain areas discussed below are regions whose functional connectivity differed as a function of emotional empathy scores within each of the four components, and because no mask or template was applied in this whole-brain analysis, individual nodes of these networks—and sometimes brain areas outside of the typical networks—may show emotional empathy-dependent variability in functional connectivity.

### 3.1. Default Mode Network

Within the DMN IC, trait empathy scores were positively correlated with the functional connectivity in the right precentral gyrus and negatively correlated with the right middle temporal gyrus, and left lingual gyrus, cuneus, and middle occipital gyrus. The cluster with its peak voxel in the precentral gyrus extended from the right premotor cortex through the right precentral and postcentral gyri (motor and somatosensory cortices, respectively).

### 3.2. Salience Network

Within the IC that contained the SN, emotional empathy scores were positively correlated with functional connectivity of the right middle temporal gyrus, and left cuneus and fusiform gyri. Negative correlations were found in relation to the functional connectivity of the right superior temporal gyrus and right inferior semi-lunar lobule of the cerebellum.

### 3.3. Left Central Executive Network

The functional connectivity of a number of structures in left CEN component correlated with emotional empathy scores. Functional connectivity in the right insula, and left cingulate and middle temporal gyri were positively correlated with IPIP scores. The functional connectivity of the right middle temporal and bilateral fusiform gyri, as well as the right culmen and left superior parietal lobule, were negatively correlated with IPIP scores.

### 3.4. Right Central Executive Network

Finally, emotional empathy correlations with functional connectivity were also observed in the right CEN. The right caudate and precuneus, and left parahippocampal gyrus were positively correlated, whereas the right culmen and superior frontal and left medial and middle frontal gyri were negatively correlated.

## 4. Discussion

The current study demonstrates that trait emotional empathy is correlated with unique patterns of functional connectivity in four of the brain’s resting state networks: the DMN, SN, right CEN, and left CEN. Importantly, many of the brain regions that showed emotional empathy-dependent variability in functional connectivity have also been linked with empathetic responses in task-based fMRI studies (e.g., [9,10]). This consistency provides support for the view that emotional empathy involves motoric, attentional, and self-referential processing.

The functional connectivity associated with the DMN in our study provides intriguing support for a motoric account of emotional empathy. In the DMN resting-state component, emotional empathy scores were positively correlated with functional connectivity in the premotor cortex and portions of the precentral (motor) and postcentral (somatosensory) cortices. It is worth noting that the premotor region is also theorized to be part of the human mirror neuron system [35,36,37] and has been found to be associated with emotional empathy ([9], although see [38] for evidence against this view). Therefore, the current data may indicate a unique link between the connectivity of sensorimotor regions (including parts of the mirror neuron system), the DMN, and trait emotional empathy. However, additional studies are necessary to more directly test this possibility.

Interestingly, the cuneus, a region implicated in task-based studies of cognitive empathy [9], was negatively correlated with trait emotional empathy in the DMN component (cuneus BA18) but positively correlated with this trait in the SN component (cuneus BA17). Additionally, the fusiform gyrus, implicated in task-based emotional empathy, was negatively correlated with the left CEN in two clusters (fusiform gyrus bilateral BA37) but again positively correlated with the SN component (fusiform gyrus BA20). The SN has been referred to as a switch between the DMN and CEN [39]. In our results, functional connectivity of two regions, the cuneus and fusiform gyrus, is positively correlated with trait emotional empathy within the IC containing the SN but negatively correlated with trait emotional empathy within the ICs containing the DMN and CEN, lending support to the concept of the SN as a neural switch.

Within the SN component, the increased functional connectivity was observed in the left cuneus (BA17) and the left fusiform gyrus (BA20). Both the cuneus and the fusiform gyrus are part of the visual system; the fusiform gyrus is particularly important for facial recognition and higher-level object representation [40]. With regards to empathy, this result is interesting as the function of the SN is to bring awareness to salient external stimuli and to integrate this information inward [22]. It is possible that an individual high in emotional empathy may have more emotion awareness [41], which would allow them to be more attuned to negative, yet salient, stimuli. Interestingly, the functional connectivity of left cuneus (BA18), middle occipital gyrus (BA19), and lingual gyrus (BA18), all components of the visual system, was negatively correlated with emotional empathy in the DMN component. Both the DMN and SN components showed a functional connectivity correlation with trait empathy for regions of the visual system. Together, these results suggest a link between empathy and sensitivity to salient environmental—and potentially social—information. However, additional behavioral testing is necessary to corroborate hypotheses based upon resting-state data [42].

The CEN is responsible for executive control [21]. Therefore, it is not surprising that it is negatively correlated with regions (i.e., the fusiform gyrus) that are related to emotional empathy. The DMN, a network that is active in the absence of a task, is negatively correlated with the cuneus (BA18), which has been found to be more active during a cognitive empathy task (BA18, [9]). This is consistent with the task-negative nature of the DMN [43].

The results of the current study are also consistent with a recent investigation of sensory processing sensitivity [44]. This trait is associated with an increased sensitivity to social stimuli, and is conceptually similar to empathy. Acevedo and colleagues [44] found that individuals with high scores on a measure of sensory processing sensitivity showed more activity in the insula, anterior cingulate, premotor area, IFG, and middle temporal gyrus when viewing photographs of loved ones. Importantly, several of these brain areas showed changed patterns of functional connectivity in the current study as well. The middle temporal gyrus (BA21, BA39) was particularly sensitive to trait emotional empathy; functional connectivity in this region was correlated with emotional empathy scores in the DMN (negative correlation with middle temporal gyrus BA39), left CEN components (negative correlation with right middle temporal gyrus BA21 and positive correlation with left middle temporal gyrus BA21), and SN (positive correlation with right middle temporal gyrus BA21). Given that activity in this region has also been linked with theory of mind (e.g., [45]), it seems reasonable to conclude that its functional connectivity is related to the ability to experience another person’s emotional states. Future research directly comparing functional connectivity and the results of task-based fMRI assessments of theory of mind would help clarify this relationship.

The results of our study indicate that brain regions characteristic of other resting state networks appeared within a different component of interest (e.g., the insula, a key node of the SN, was positively correlated with empathy scores within the left CEN component). These findings are interesting as previous research has revealed similar relationships between networks and network nodes. The observation of regions of one network showing functional connectivity with another network has been shown [46] and observations of relationships between networks have been reported [47]. Although we did not assess the relationship between networks, our investigation within each network suggests that the functional connectivity within resting state networks can involve key nodes of other networks, at least in the context of a correlation with trait empathy. For example, in the DMN component, the empathy scores are positively correlated with the functional connectivity of the right precentral gyrus (BA6), a key node of the sensorimotor network [30]. The left CEN demonstrated increased functional connectivity with the right insula (BA13) and cingulate gyrus (BA24) (belonging to the SN) and changes in functional connectivity within bilateral middle temporal gyri (BA21, component of a language network [30]. In the right CEN, increased functional connectivity was observed in the precuneus (BA31) and superior frontal (BA10), middle frontal (BA10), and medial frontal (BA9) gyri—precuneus and prefrontal cortices are key regions of the DMN. Gaining a further understanding of how trait empathy is related to inter-network connectivity could enhance our understanding of several clinical conditions.

The above findings should be considered along with study limitations. First, it is important to note that due to the nature of the analyses used, the findings of this study do not represent causal relationships. That is, the results represent a correlational relationship between a questionnaire-based measure of emotional empathy and brain functional connectivity. Second, no control network was used, and this has limited our ability to assess specificity. Third, we were also limited in examining only emotional empathy and not cognitive empathy. It would be important for future research to examine the resting state neural correlates of cognitive empathy to provide the most comprehensive understanding of the neural representation of empathy. Fourth, due to the imbalance in sample size of men and women, we were unable to consider gender as a factor in our analyses. Considering the differential findings of emotional empathy between men and women [48], future research should be conducted. Similarly, in using an undergraduate sample, the generalizability of these findings is limited.

## 5. Conclusions

These limitations notwithstanding, the current research provides novel information about the empathetic brain. In contrast to previous studies which used structural MRI or task-based fMRI to assess the biology of empathy, the current research used an ICA-based analysis of resting brain activity. This “data driven” approach allowed us to measure emotional empathy–dependent changes in functional connectivity across the brain without being limited to specific seed regions. The fact that the current resting-state fMRI results largely overlap with previous task-based studies helps to corroborate our findings. It also suggests that the individual differences found in task-based studies may be driven by individual differences in trait emotional empathy.

## Figures and Tables

**Table 1:** Brain regions showing correlation of trait empathy questionnaire scores with functional connectivity values within the Default Mode Network, Salience Network, left Central Executive Network, and right Central Executive Network components. Talairach coordinates for the peak voxel in each cluster are given. Right (R) and left (L) sides of the brain are indicated; BA denotes Brodmann Area, if applicable, and the number of voxels in each of the clusters with corresponding *r*- and *p*-values.

Region	Side	BA	Talairach Coordinates			Voxels	*r*	*p*
			X	Y	Z			
**Default Mode Network**								
Middle Temporal Gyrus	R	39	51	−67	13	1896	−0.546	0.001
Precentral Gyrus	R	6	33	−13	64	8000	0.635	0.000
Lingual Gyrus	L	18	−3	−98	−11	3082	−0.653	0.000
Cuneus	L	18	−6	−97	23	2041	−0.552	0.001
Middle Occipital Gyrus	L	19	−24	−82	16	2031	−0.460	0.008
**Salience Network**								
Superior Temporal Gyrus	R	22	69	−36	7	3025	−0.542	0.001
Middle Temporal Gyrus	R	21	48	5	−17	5714	0.567	0.001
Inferior Semi-Lunar Lobule	R		6	−76	−41	6723	−0.500	0.004
Cuneus	L	17	−9	−85	7	6547	0.669	0.000
Fusiform Gyrus	L	20	−51	−34	−26	2968	0.532	0.002
**Left Central Executive Network**								
Middle Temporal Gyrus	R	21	63	−25	−14	4074	−0.601	0.000
Insula	R	13	48	−25	19	4865	0.590	0.000
Fusiform Gyrus	R	37	48	−61	−17	3185	−0.516	0.002
Culmen	R		3	−37	−2	14,385	−0.622	0.000
Cingulate Gyrus	L	24	−6	−19	34	8083	0.615	0.000
Superior Parietal Lobule	L	7	−27	−73	43	4441	−0.531	0.002
Middle Temporal Gyrus	L	21	−57	−1	−30	9185	0.630	0.000
Fusiform Gyrus	L	37	−48	−58	−17	3476	−0.597	0.000
**Right Central Executive Network**								
Superior Frontal Gyrus	R	10	30	59	−8	6209	−0.605	0.000
Culmen	R		24	−61	−26	22,335	−0.606	0.000
Caudate Body	R		21	20	13	5887	0.537	0.002
Precuneus	R	31	9	−61	19	16,340	0.661	0.000
Medial Frontal Gyrus	L	9	−12	26	31	6315	−0.512	0.003
Medial Frontal Gyrus	L	6	−6	−10	61	4362	−0.581	0.000
Parahippocampal Gyrus	L	35	−21	−25	−20	5999	0.618	0.000
Middle Frontal Gyrus	L	10	−27	56	−8	5987	−0.553	0.001

## Supplementary Materials

The following are available online at http://www.mdpi.com/2076-3425/8/7/128/s1, Table S1: Psychometric characteristics of the emotional empathy measure used in the current sample and comparative psychometric data from a second, independent sample; Table S2: Conjoint factor analysis of emotional empathy items used in the current study with established trait indicators of emotional and cognitive empathy.
