# Supplementary material for: Trait Emotional Empathy and Resting State Functional Connectivity in Default Mode, Salience, and Central Executive Networks
Source: Brain Sci. 2018 Jul 6;8(7):128. doi: 10.3390/brainsci8070128 (PMC6071260; doi:10.3390/brainsci8070128)
Supplement: Supplementary file 1 [file brainsci-08-00128-s001.pdf]

Supplementary Table S1. Psychometric characteristics of the emotional empathy measure used in the current sample and comparative psychometric data from a second, independent sample.

			Total			Women			Men			
Sample	α	$\overline{r}_{ii}$	N	Mn	SD	N	Mn	SD	N	Mn	SD	d
MRI sample	.84	.48	31	3.63	.79	21	3.73	.76	10	3.42	.86	.39
Remainder sample	.76	.35	1,863	3.55	.66	1352	3.63	.65	511	3.34	.65	.44
Comparison sample												
Emo. empathy	.79	.38	739	3.61	.65	519	3.68	.61	220	3.44	.70	.37
Empathic concern	.78	.34	739	3.79	.62	519	3.90	.60	220	3.56	.62	.58
Perspective-taking	.80	.37	739	3.53	.68	519	3.52	.67	220	3.53	.71	.13

Note: Data in the first two rows are for the six-item emotional empathy scale used in the current samples. Data in the bottom three rows are from an unpublished comparison sample collected several years prior to the current samples, from which data were available for the six-item emotional empathy scale used in the current study as well two other trait empathy measures, the Davis (1980) Empathic Concern and Perspective-taking scales; all three measures had been administered with a similar 5-place response scale. Column labels are Coefficient Alpha (α), mean inter-item correlation ($\overline{r}_{ii}$), sample mean (Mn), sample standard deviation (SD), gender difference effect size (d).

Supplementary Table S2. Conjoint factor analysis of emotional empathy items used in the current study with established trait indicators of emotional and cognitive empathy.[1]

	Factor[2]		
Measure	I	II	II
Emotional Empathy Scale items			
Feels others emotions.	-.02	***.72***	-.03
Suffers from others sorrows.	-.11	***.62***	.14
Anticipates the needs of others.	.13	***.51***	-.07
Is deeply moved by others misfortunes.	-.03	***.45***	.20
Senses others wishes.	.08	***.63***	-.19
Is very empathic, intensely feels what others feel.	-.03	***.69***	.08
IRI Empathic Concern Scale items			
I often have tender, concerned feelings for people less fortunate than me.	-.01	.21	***.43***
Sometimes I don't feel very sorry for other people when they are having problems.	-.02	-.16	***.70***
When I see someone being taken advantage of, I feel kind of protective towards them.	.16	.03	***.29***
Other peoples' misfortunes do not usually disturb me a great deal.	-.03	.07	***.73***
When I see someone being treated unfairly, I sometimes don't feel very much pity for them.	.12	-.12	***.61***
I am often quite touched by things that I see happen.	.07	.27	***.35***
I would describe myself as a pretty soft-hearted person.	-.01	.25	***.40***
IRI Perspective-Taking Scale items			
Before criticizing somebody, I try to imagine how I would feel if I were in their place.	***.60***	.05	.05
When I'm upset at someone, I usually try to "put myself in his shoes" for awhile.	***.56***	.11	-.02
I try to look at everybody's side of a disagreement when I make a decision.	***.76***	-.08	-.01
I sometimes find it difficult to see things from the "other guy's" point of view.	***.45***	-.04	.12
If I'm sure I'm right about something, I don't waste much time listening to other people's arguments.	***.48***	-.12	.12
I believe that there are two sides to every question and I try to look at them both.	***.74***	.01	-.05
I sometimes try to understand my friends better by imagining how things look from their perspective.	***.60***	.16	-.06

Note: Comparison sample, *N*=739. [1] The Perspective-Taking and Empathic Concern subscales, respectively, of the Interpersonal Reactivity Index (IRI; Davis, 1980). [2]Shown are rotated pattern matrix loadings in a three factor solution to intercorrelations among responses to these 20 empathy items (Principal Factor extraction, Promax rotation); loadings > |.30| are presented bolded italic font; the first six eigenvalues in this analysis (and percent of variance explained prior to rotation) were 6.0 (29.9%), 2.2 (11.0%), 1.3 (6.4%), 1.1 (5.5%), 1.1 (5.4%), and 1.0 (4.8%); factor correlations, in order of magnitude, were .67 (II, III), .49 (II, I), and .35 (I, III).
